# Common risk variants for colorectal cancer: an evaluation of associations with age at cancer onset

**DOI:** 10.1038/srep40644

**Published:** 2017-01-13

**Authors:** Nan Song, Aesun Shin, Ji Won Park, Jeongseon Kim, Jae Hwan Oh

**Affiliations:** 1 Cancer Research Institute, Seoul National University College of Medicine, Seoul, 03080, Korea; 2 Department of Preventive Medicine, Seoul National University College of Medicine, Seoul, 03080, Korea; 3 Molecular Epidemiology Branch, National Cancer Center, Goyang, 10408, Korea; 4 Department of Surgery, Seoul National University College of Medicine and Hospital, Seoul, 03080, Korea; 5 Center for Colorectal Cancer, National Cancer Center, Goyang, 10408, Korea

## Abstract

Common genetic risk variants for colorectal cancer (CRC) have been identified at approximately 40 loci by genome-wide association studies (GWAS). We investigated the association of these risk variants by age at onset of CRC using case-only and case-control analysis. A total of 1,962 CRC cases and 2,668 controls from two independent case-control studies conducted by Korea’s National Cancer Center were included in this study. We genotyped 33 GWAS-identified single-nucleotide polymorphisms (SNPs) associated with CRC risk. The risk allele in SNP rs704017, located at 10q22.3 in the *ZMIZ1-AS1* gene, was consistently less frequent among CRC patients aged <50 years than among CRC patients aged ≥50 years in the case-only analysis (odds ratio (OR) = 0.78, 95% confidence interval (CI) = 0.66–0.92, *P* = 2.7 × 10^−3^, in an additive model), although this did not surpass the threshold for multiple testing. The direction of associations between rs704017 and CRC risk differed by age group in the combined case-control analysis (<50 years: OR = 0.77, 95% CI = 0.60–0.98, *P* = 0.03 and ≥50 years: OR = 1.13, 95% CI = 0.98–1.29, *P* = 0.09, in a dominant model); the *p*-values for heterogeneity (*P*_heterogeneity_ = 7.5 × 10^−3^) and for interaction were statistically significant (*P*_interaction_ = 7.8 × 10^−3^, in the dominant model). Our results suggest that the CRC susceptibility SNP rs704017 has a hereditary effect on onset age of CRC.

Hereditary factors are thought to contribute about 35% to causation of colorectal cancer (CRC)^1^. This view is supported by the fact that while rare genetic variants with high penetrance do confer a predisposition for inherited forms of CRC, such as the *APC* gene mutation in familial adenomatous polyposis (FAP) and the mismatch repair (MMR) gene mutation in Lynch syndrome, they account for only about 5% of CRC cases^2^. In order to explain the remaining genetic heritability, genome-wide association studies (GWAS) have identified approximately 40 common genetic loci for sporadic CRC^3^; susceptibility single-nucleotide polymorphisms (SNPs) are thought to confer weak but cumulative and increasing effects on CRC risk^4^.

Genetic variants in susceptibility SNPs for CRC are likely to influence age at onset^4^. It has been suggested that, compared with late-onset CRC, the genetic contributions are enriched in early-onset CRC^5^ in that clinico-pathologically advanced disease and poor prognosis^6^. Furthermore, the fact that age was differently distributed according to molecular features, such as CpG island methylator phenotype (CIMP)^7^, DNA macrosatellite instabilitly (MSI) status^8^, precursor adenomas^9^, and mutations in *BRAF* or *KRAS* gene^9^ in sporadic CRC suggests that a distinct genetic background contributes to the disease that differs between early- and late-onset CRC^4^. Furthermore, a considerable number of unidentified genetic variants remain and replication studies of previously reported CRC susceptibility SNPs according to age at onset are needed.

We hypothesized that several common genetic variants of susceptibility SNPs could be related either to early or late age at onset of CRC. To test this hypothesis, allele frequencies of 33 susceptibility SNPs identified by previous GWAS were compared between early-onset CRC patients (aged <50 years) and later-onset CRC patients (aged ≥50 years) in a case-only analysis. We assessed the heterogeneity of associations between SNPs and CRC risk according to age groups and interactions between SNPs and age groups in case-control analyses.

## Results

Table 1 shows the baseline characteristics of CRC patients in each study. A total of 1,962 sporadic CRC patients comprising 436 early-onset (aged <50 years, mean: 42.5 years) patients and 1,526 late-onset (aged ≥50 years, mean: 62.2 years) patients were included in this analysis. In both the NCC 2010–2013 and NCC 2000–2004 studies, late-onset CRC patients were more likely to have higher body mass index (*P*_NCC 2010–2013_ = 0.03, *P*_NCC 2000–2004_ < 0.01, and *P*_combined_ < 0.01) and a lower education level (*P*_NCC 2010–2013_ < 0.01, *P*_NCC 2000–2004_ < 0.01, and *P*_combined_ < 0.01) than early-onset patients. Early-onset patients were more likely than late-onset patients to have reported ever consuming alcohol (*P*_NCC 2000–2004_ < 0.01 and *P*_combined_ < 0.01). In the NCC 2010–2013, late-onset CRC patients had more frequency of fecal occult blood test (FOBT) history than early-onset patients. There were no differences for sex, smoking, TNM stage, or CRC site between onset age groups.

All SNPs were in HWE (*P* > 0.05) except for one SNP, rs10411210 on 19q13.11 (*RHPN2, P* = 0.01). When CRC patients aged ≥50 years were considered as the reference, the additive risk allele (G) of SNP rs704017 at 10q22.3 (*ZMIZ1-AS1*) was less frequent among patients aged <50 years (OR_NCC 2010–2013_ = 0.72, 95% CI = 0.54–0.97, *P* = 0.03, OR_NCC 2002–2004_ = 0.80, 95% CI = 0.66–0.98, *P* = 0.03, and OR_combined_ = 0.78, 95% CI = 0.66–0.92, *P* = 2.7 × 10^−3^) (Table 2). When late-onset CRC patients were restricted to patients aged ≥65 years for the sensitivity analysis (Supplementary Table 2), the RAF of rs704017 also tended to be less frequent among early-onset patients (OR_NCC 2010–2013_ = 0.63, 95% CI = 0.44–0.90, *P* = 0.01, OR_NCC 2002–2004_ = 0.80, 95% CI = 0.64–1.01, *P* = 0.06, and OR_combined_ = 0.63, 95% CI = 0.63–0.93, *P* = 6.6 × 10^−3^). No other SNPs showed differences in RAFs between onset age groups (*P* > 0.05).

In the NCC 2010–2013 study, rs704017 was significantly associated with increased risk of CRC among patients aged ≥50 years (OR_additive model_ = 1.24, 95% CI = 1.06–1.45, *P* = 6.5 × 10^−3^ and OR_dominant model_ = 1.42, 95% CI = 1.14–1.77, *P* = 2.0 × 10^−3^) with significant heterogeneity for associations between age groups (*P*_additive model_ = 0.04 and *P*_dominant model_ = 0.02) and genotype × age group interaction (*P*_additive model_ = 0.04 and *P*_dominant model_ = 0.02) (Table 3). Although no significant association was observed in the NCC 2000–2004 study, rs704017 was associated with decreased risk for CRC among patients aged <50 years in the combined dataset (OR_dominant model_ = 0.77, 95% CI = 0.60–0.98, *P* = 0.03) and the combined associations were in the opposite direction among patients aged ≥50 years in age. In addition, we found heterogeneity between age groups (*P*_additive model_ = 0.02 and *P*_dominant model_ = 7.5 × 10^−3^) and interaction between genotypes and age groups (*P*_additive model_ = 0.02 and *P*_dominant model_ = 7.8 × 10^−3^) in the combined dataset.

## Discussion

We found that the risk allele of SNP rs704017 at 10q22.3 (*ZMIZ1-AS1*) was less frequent among sporadic CRC patients with an early age at onset (<50 years) than among patients with late-onset age (≥50 years) in our case-only analysis. Furthermore, both heterogeneity and interaction was observed in the association between genotypes of rs704017 and risk for CRC according to age groups (<50 and ≥50 years) in our case-control analysis.

Early-onset CRC includes approximately 30% of hereditary and 70% of sporadic CRC cases^10^. The molecular mechanisms driving hereditary early-onset CRC have been well defined as germline mutations such as the *MLH1, MSH2, MSH6, PMS2*, and *EPCAM* mutations in Lynch syndrome and *APC* and *MUTYH* mutations in FAP^11^, whereas sporadic early-onset CRC has not been fully clarified^10^. Although sporadic early-onset CRC is thought to be attributable to common genetic variants with low penetrance^4^, only a few SNPs, including rs10795668 at 10p14, rs3802842 at 11q23.1, and rs4779584 at 15q13.3, have been associated with an increased risk for early-onset CRC^12^.

We found that the risk allele (G) of rs704017 was less frequent among early-onset CRC patients and was associated with increased risk among late-onset CRC patients. Accordingly, it may be that this variant plays a role in genetic predisposition to late-onset CRC. To date, a few associations of this risk variant for CRC have been reported among East Asians (*P* = 2.07 × 10^−8^) and Europeans (*P* = 4.71 × 10^4^)^13^. In those analyses, the mean age of CRC patients was 60.25 years in East Asians and 64.10 years in Europeans, and the analyses included all CRC patients regardless of onset age. Therefore, there is a need for more association studies, in order to confirm the associations of rs704017 with CRC risk according to onset age.

Rs704017 is located in an intron of the zinc finger MIZ-type containing 1 antisense RNA1 (*ZMIZ1-AS1*) gene in the 10q22.3 region. *ZMIZ1-AS1* interferes with and inhibits translation of *ZMIZ1* gene. Reduced *ZMIZ1* gene expression and greater frequencies of somatic mutations were observed in colon tumors based on data from The Cancer Genome Atlas (TCGA)^14^ and the Catalogue of Somatic Mutation in Cancer (COSMIC)^15^. The *ZMIZ1* gene encodes a part of the protein inhibitor of activated signal transducer and activator of transcription (STAT) protein family (PIAS). With a Janus kinase (JAK), the STAT protein belongs to JAK-STAT signaling pathway, which can control survival, proliferation, and differentiation of various cells^16^. The oncogenic transformation can be promoted by persistently activated STAT proteins because of several somatic mutations in the JAK-STAT pathway, which have been identified in patients with a variety of diseases, including myeloproliferative disease, polycythemia vera, megakaryoblastic myeloid leukemia, lymphoblastic leukemia, and uterine leimyosarcomas^16^, and also could cause CRC^17^.

A large proportion of CRC patients have late-onset sporadic disease without an obvious hereditary syndrome^18^. Although the majority of late-onset CRC is located in the distal colon and microsatellite stable (MSS), some features more characteristic of late-onset CRC include occurrence in the proximal colon, as well as the presence of MSI via *MLH1* gene promoter methylation, chromosomal instability, and a high CpG island methylator phenotype, especially when compared with sporadic early-onset CRC^11^. In addition to these characteristics, constitutively decreased PTEN expression in colon mucosa and p53 were experimentally observed to be associated with a late process of tumorigenesis in CRC^19^,^20^,^21^. Because the PIAS protein family has been known to regulate p53^22^ and PTEN^23^, tumor development of CRC may also occur late.

On the other hand, rs704017 (G) was less frequent and tended to be associated with decreased risk of early-onset CRC compared to late-onset CRC. This is because rs704017 might have only small effects on early-onset CRC according to both the common disease-common variant hypothesis^24^ and the polygenic inheritance model^25^. Moreover, several early-onset sporadic CRC cases without family history showed the possibility of hereditary CRC suggesting a role for germline mutations in *hMLH1* and *hMSH2* in carcinogenesis in contrast to general sporadic CRC, which is more related to epigenetic changes^26^. Thus, tumorigenesis of early-onset CRC could be more influenced by germline mutators than by somatic mutations.

An age of 50 years has been considered the cut-off for early- vs. late-onset CRC according to previous publications^11^,^27^. The reason that CRC screening is recommended for people starting at age 50 years in Korea^28^, as well as in many other national guidelines^29^,^30^,^31^, is because screening colonoscopy studies have shown a significantly increased risk of advanced neoplasms among people older than 50 years^32^,^33^,^34^. Additionally, we considered CRC patients aged 65 years or more as late-onset for the sensitivity analysis. We also compared allelic frequencies between CRC patients aged under 30 or 40 (early-onset) and patients aged 50 or 65 years or more (late-onset), but the results were more attenuated due to small sample size effects.

One strength of our study is that we evaluated the association of risk variants according to onset age of CRC throughout both stages of our case-only and case-control analyses. Because case-only analysis is considered to produce more precise estimations than case-control analysis due to both small dispersion and homogeneity^35^, we conducted a case-only analysis before the case-control analysis. From those analyses, we were able to observe the relationship between rs704017 and onset age of CRC. A limitation of this study is that although we made adjustments for multiple testing, specifically the Bonferroni and false-discovery rate (FDR) tests, the association of rs704017 with CRC onset age was not statistically significant. The *p*-value was 2.7 × 10^−3^ in the combined dataset when comparing allele frequencies between onset age of CRC patients. However, *p*-values were compared to 0.05 divided by 33 (=1.5 × 10^−3^) which was the Bonferroni-corrected *p*-value for 33 SNPs and the FDR-adjusted *p*-value of rs704017 was estimated to be 0.09. Accordingly, adjustments for multiple testing were not applied to the results and further analyses with larger sample sizes are needed to prevent false-positive results and confirm the possible association noted in this study.

In conclusion, we found that the risk variant of rs704017 at 10q22.3 (*ZMIZ1-AS1*) was significantly less frequent among early-onset sporadic CRC patients, although this did not surpass the threshold for multiple testing. Moreover, the association between rs704017 and risk of CRC tended to be in opposite directions according to the onset age, and heterogeneity and genotype-onset age interaction were observed. To ascertain the role of susceptibility SNPs on the onset age of CRC, further studies are needed.

## Methods

### Study population

This study used data from two independent, hospital-based case-control studies conducted by the National Cancer Center (NCC) in Korea, NCC 2010–2013 and NCC 2000–2004, the details of which have been reported previously^13^,^36^,^37^. NCC 2010–2013 recruited 1,070 newly diagnosed CRC patients, who had been surgically treated between 2010 and 2013. The controls were recruited from among people who visited a cancer-screening center at the NCC for a health check-up through a benefit program of the National Health Insurance Corporation between 2007 and 2014. After excluding individuals who did not complete a structured written questionnaire or whose blood sample was insufficient for genotyping, the remaining 703 cases were 1:2 matched with 1,406 controls by sex and age (5-year intervals). Of these, 49 cases and 67 controls who had a first- or second-degree family history of CRC were also excluded. Thus, a total of 654 cases and 1,339 controls from NCC 2010–2013 were included in the analysis.

In NCC 2000–2004, cases comprised CRC patients who had been histologically confirmed and received surgical treatment between 2000 and 2004 at the same hospital as NCC 2010–2013. After applying the same exclusion criteria used in NCC 2010–2013, a total of 1,308 sporadic CRC patients were eligible for the analysis. Among controls who were recruited from the same cancer-screening center as NCC 2010–2013 between 2002 and 2004, 1,329 individuals were frequency-matched with cases by age and sex for NCC 2000–2004. All participants were mutually exclusive between the two studies. All study participants provided the written informed consent to participate. Both studies were approved by the institutional review board (IRB) of the NCC (IRB No. NCCNCS-10-350 and NCCNCS-10-396).

### Data collection

From CRC patients, general and lifestyle information on age, sex, body mass index, education level, alcohol consumption and smoking habits, and previous FOBT history was obtained by a face-to-face interview conducted by a trained interviewer using a structured, written questionnaire. Clinico-pathological information on tumor-node-metastasis (TNM) stage and CRC site was obtained from patients’ medical records from the Center for Colorectal Cancer at the NCC. The control participants conducted self-administered questionnaires on general and lifestyle information, after which an interviewer contacted them by phone and confirmed the participants’ responses.

### Genotyping

For genotyping, we selected 36 susceptibility SNPs at 27 loci that had been associated with CRC risk by previous GWAS^13^,^38^,^39^,^40^,^41^,^42^,^43^,^44^,^45^,^46^,^47^. For participants in the NCC 2010–2013 study, genomic DNA from blood was extracted using the MagAttract DNA Blood M48 kit and BioRobot M48 automatic extraction equipment (Qiagen, Inc., Valencia, CA, US), according to the manufacturer’s instructions. Genotyping was performed using Agenabio MassArray iPLEX^®^ gold assay (Agena Bioscience, Inc., San Diego, CA, US), and 32 of the 36 selected SNPs (88.9%) were successfully genotyped. Genotyping for the NCC 2000–2004 study had been conducted using the iPLEX Sequenom MassARRAY platform (Sequenom, Inc., San Diego, CA, US) for 29 susceptibility SNPs as previously described^13^,^37^, and 28 SNPs overlapped with the 36 SNPs selected for this analysis. Accordingly, one SNP rs719725 was excluded from the two studies. Additionally, two SNPs, rs6691170 and rs16892766, were monomorphic and therefore excluded. Thus, of the originally selected 36 SNPs, a total of 33 GWAS-identified SNPs at 25 loci were included in the analysis (Supplementary Table 1). All experimental methods were approved by the IRB of the NCC and performed in accordance with the manufacturer acguidelines and regulations.

### Statistical analysis

To compare the characteristics of sporadic CRC patients aged <50 years with those aged ≥50 years, we used Student’s t-test for continuous variables and the chi-square test for categorical variables. For all selected SNPs, Hardy-Weinberg Equilibrium (HWE) was tested among controls. The risk allele frequencies (RAFs) of the SNPs were calculated for each early-onset (aged <50 years) and late-onset (aged ≥50 years) CRC patient and all controls. To compare the RAFs of SNPs between onset age groups (<50 and ≥50 years) of the CRC patients under an additive model, a logistic regression model adjusted for sex was used. For the sensitivity analysis, the RAFs of SNPs were also compared between CRC patients aged <50 years and those aged ≥65 years. To investigate associations of susceptibility SNPs with CRC risk according to age groups (<50 and ≥50 years) under additive, dominant, and recessive models, we used logistic regression models adjusted for age and sex and stratified by age groups. The heterogeneity of the associations between age groups was evaluated with Cochran’s Q test. Interactions were assessed with Wald statistics by adding a genotype × age group interaction term to the models. The heterogeneity of the association for SNP, rs704017, between NCC 2010–2013 and NCC 2000–2004 was evaluated with Cochran’s Q test and random effect meta-analysis as well as pooled analysis was performed. Since there was not statistically significant heterogeneity between two study groups and showed similar combined results, the pooled analysis was applied in combined results of two study groups. Because of multiple comparison problems, Bonferroni and the false-discovery rate (FDR) tests were conducted. Associations were evaluated by odds ratios (ORs) and 95% confidence intervals (CIs), and *p*-values less than 0.05 were considered to be statistically significant. All statistical analyses were two-sided and performed separately in each of the datasets from the two studies and in the combined dataset using SAS version 9.3 software (SAS Institute, Inc., Cary, NC, US) and STATA version 13 software (STATA Corp., College Station, TX, US).

## Additional Information

**How to cite this article:** Song, N. *et al*. Common risk variants for colorectal cancer: an evaluation of associations with age at cancer onset. *Sci. Rep.* **7**, 40644; doi: 10.1038/srep40644 (2017).

**Publisher's note:** Springer Nature remains neutral with regard to jurisdictional claims in published maps and institutional affiliations.

## Supplementary Material

Supplementary Tables

## Figures and Tables

**Table 1:** Characteristics of colorectal cancer patients by age of onset.

Characteristics	NCC (2010–2013)							NCC (2000–2004)						
			Age of onset				*P*^a^			Age of onset				*P*^a^
	Total							Total		<50 years		<50 years			<50 years		<50 years	
	(N = 654)		(N = 140)		(N = 514)			(N = 1,308)		(N = 296)		(N = 1,012)				
	N	(%)	N	(%)	N	(%)		N	(%)	N	(%)	N	(%)			
Age (years), mean (SD)	56.6	(9.4)	43.1	(5.1)	60.2	(6.6)	<0.01	58.5	(11.4)	42.2	(5.8)	63.3	(7.7)	<0.01
Sex							0.11							0.23
Male	439	(67.1)	86	(61.4)	353	(68.7)		817	(62.5)	176	(59.5)	641	(63.3)	
Female	215	(32.9)	54	(38.6)	161	(31.3)		491	(37.5)	120	(40.5)	371	(36.7)	
BMI (kg/m^2^), mean (SD)	23.8	(3.4)	23.2	(3.8)	24.0	(3.3)	0.03	23.4	(3.1)	22.9	(3.0)	23.6	(3.1)	<0.01
Education							<0.01							<0.01
≤Middle school	241	(36.9)	14	(10.0)	227	(44.2)		504	(38.5)	55	(18.6)	449	(44.4)	
High school	248	(37.9)	60	(42.9)	188	(36.6)		330	(25.2)	109	(36.8)	221	(21.8)	
≥College	165	(25.2)	66	(47.1)	99	(19.3)		263	(20.1)	96	(32.4)	167	(16.5)	
Alcohol drinking							0.22							<0.01
Never	196	(30.0)	36	(25.7)	160	(31.1)		632	(48.3)	122	(41.2)	510	(50.4)	
Ever	458	(70.0)	104	(74.3)	354	(68.9)		651	(49.8)	169	(57.1)	482	(47.6)	
Smoking							0.41							0.05
Never	293	(44.8)	67	(47.9)	226	(44.0)		707	(54.1)	146	(49.3)	561	(55.4)	
Ever	361	(55.2)	73	(52.1)	288	(56.0)		576	(44.0)	145	(49.0)	431	(42.6)	
TNM stage							0.08							0.96
≤Stage I	170	(26.0)	26	(18.6)	144	(28.0)		180	(13.8)	41	(13.9)	139	(13.7)	
Stage II	148	(22.6)	29	(20.7)	119	(23.2)		353	(27.0)	78	(26.4)	275	(27.2)	
Stage III	231	(35.3)	54	(38.6)	177	(34.4)		538	(41.1)	122	(41.2)	416	(41.1)	
Stage IV	78	(11.9)	22	(15.7)	56	(10.9)		224	(17.1)	54	(18.2)	170	(16.8)	
CRC site							0.61							0.14
Colon	325	(49.7)	71	(50.7)	254	(49.4)		646	(49.4)	136	(46.0)	510	(50.4)	
Rectum	312	(47.7)	63	(45.0)	249	(48.4)		649	(49.6)	159	(53.7)	490	(48.4)	
FOBT history							<0.01							—
Never	369	(56.5)	126	(90.0)	243	(47.4)		—		—		—		
Ever	284	(43.5)	14	(10.0)	270	(52.6)		—		—		—		

Abbreviations: NCC (National Cancer Center), SD (standard deviation), BMI (body mass index), TNM (tumor-node-metastasis), CRC (colorectal cancer), FOBT (fecal occult blood test).

^a^T-test for continuous variables and chi-square test for categorical variables between CRC patients with <50 and ≥50 years.

**Table 2:** Allelic frequency comparison of identified susceptibility single-nucleotide polymorphisms between age of onset groups (<50 vs. ≥50 years) in colorectal cancer patients.

SNP	Cytogenetic region	Mapped gene			NCC 2010–2013					NCC 2000–2004					Combined dataset				
			Allele^a^		RAF by age		OR^b^	95% CI	*P*^c^	RAF by age		OR^b^	95% CI	*P*^c^	RAF by age		OR^b^	95% CI	*P*^c^
			A1	A2	<50	≥50				<50	≥50				<50	≥50			
rs6687758	1q41	*intergenic*	G	A	0.27	0.31	0.86	0.63–1.19	0.36	0.30	0.29	1.06	0.86–1.30	0.61	0.29	0.30	0.99	0.83–1.18	0.92
rs10936599	3q26.2	*MYNN*	T	C	0.62	0.61	1.01	0.77–1.34	0.92	0.59	0.59	1.02	0.85–1.23	0.83	0.61	0.60	1.02	0.87–1.19	0.83
rs647161	5q31.1	*C5orf66*	A	C	0.37	0.33	1.19	0.90–1.58	0.22	0.34	0.34	1.02	0.84–1.23	0.84	0.35	0.34	1.07	0.92–1.25	0.40
rs7758229	6q25.3	*SLC22A3*	T	G	0.24	0.22	1.07	0.79–1.46	0.66	0.20	0.22	0.89	0.71–1.11	0.29	0.21	0.22	0.95	0.79–1.13	0.54
rs6983267	8q24.21	*CASC8, CCAT2*	T	G	0.50	0.53	0.89	0.68–1.16	0.37	0.52	0.55	0.90	0.75–1.09	0.28	0.52	0.54	0.90	0.77–1.04	0.16
rs7014346	8q24.21	*CASC8*	G	A	0.66	0.68	0.92	0.69–1.21	0.54	—	—	—	—	—	0.66	0.68	0.92	0.69–1.21	0.54
rs10505477	8q24.21	*CASC8*	G	A	0.50	0.54	0.87	0.67–1.14	0.32	—	—	—	—	—	0.50	0.54	0.87	0.67–1.14	0.32
rs10795668	10p14	*LOC105376400*	A	G	0.36	0.34	1.07	0.81–1.40	0.64	0.35	0.34	1.02	0.84–1.23	0.86	0.35	0.34	1.04	0.89–1.21	0.67
rs704017^d^	10q22.3	*ZMIZ1-AS1*	G	A	0.32	0.39	0.72	0.54–0.97	0.03	0.29	0.34	0.80	0.66–0.98	0.03	0.30	0.36	0.78	0.66–0.92	2.7 × 10^−3^
rs11196172	10q25.2	*TCF7L2*	A	G	0.78	0.76	1.13	0.82–1.57	0.46	0.74	0.74	0.96	0.80–1.21	0.89	0.75	0.75	1.02	0.86–1.22	0.79
rs1665650	10q25.3	*HSPA12A*	C	T	0.66	0.69	0.89	0.66–1.17	0.37	0.66	0.66	1.03	0.85–1.25	0.79	0.66	0.67	0.98	0.83–1.15	0.77
rs174537	11q12.2	*MYRF*	T	G	0.27	0.29	0.92	0.68–1.25	0.59	0.28	0.29	0.91	0.74–1.12	0.39	0.28	0.29	0.92	0.77–1.09	0.32
rs4246215	11q12.2	*FEN1*	T	G	—	—	—	—	—	0.28	0.30	0.90	0.73–1.11	0.31	0.28	0.30	0.90	0.73–1.11	0.31
rs174550	11q12.2	*FADS1*	T	C	0.72	0.70	1.07	0.79–1.44	0.66	0.72	0.70	1.10	0.89–1.35	0.39	0.72	0.70	1.09	0.92–1.29	0.34
rs1535	11q12.2	*FADS2*	A	G	0.72	0.71	1.07	0.79–1.45	0.66	0.72	0.70	1.11	0.90–1.37	0.32	0.72	0.70	1.10	0.93–1.30	0.29
rs3802842	11q23.1	*COLCA1, COLCA2*	A	C	0.58	0.59	0.98	0.74–1.30	0.89	0.59	0.59	1.03	0.86–1.24	0.76	0.59	0.59	1.02	0.87–1.19	0.84
rs10849432	12p13.31	*intergenic*	T	C	0.80	0.84	0.74	0.53–1.02	0.07	0.84	0.83	1.09	0.85–1.39	0.51	0.83	0.84	0.95	0.78–1.16	0.61
rs10774214	12p13.32	*CCND2-AS1*	C	T	0.56	0.57	0.97	0.74–1.28	0.84	0.54	0.55	0.94	0.78–1.13	0.48	0.54	0.56	0.95	0.81–1.10	0.48
rs11169552	12q13.12	*ATF1, LOC105369765*	T	C	0.29	0.34	0.79	0.59–1.06	0.11	0.34	0.47	1.08	0.89–1.31	0.43	0.33	0.33	0.98	0.84–1.15	0.81
rs7136702	12q13.13	*intergenic*	C	T	0.43	0.49	0.79	0.61–1.04	0.09	0.49	0.47	1.06	0.88–1.27	0.54	0.47	0.48	0.97	0.83–1.12	0.65
rs4444235	14q22.2	*intergenic*	C	T	0.55	0.52	1.15	0.88–1.50	0.30	0.55	0.55	1.01	0.84–1.22	0.90	0.55	0.54	1.05	0.91–1.23	0.49
rs1957636	14q22.3	*LOC105370507*	C	T	0.41	0.43	0.91	0.69–1.19	0.47	—	—	—	—	—	0.41	0.43	0.91	0.69–1.19	0.47
rs4779584	15q13.3	*intergenic*	C	T	0.12	0.14	0.86	0.57–1.28	0.45	0.16	0.16	1.04	0.81–1.33	0.78	0.15	0.15	0.98	0.80–1.22	0.87
rs9929218	16q22.1	*CDH1*	A	G	0.14	0.17	0.80	0.55–1.16	0.25	0.13	0.15	0.88	0.67–1.16	0.37	0.14	0.15	0.85	0.68–1.06	0.15
rs12603526	17p13.3	*intergenic*	C	T	0.35	0.36	0.99	0.75–1.32	0.96	—	—	—	—	—	0.35	0.36	0.99	0.75–1.32	0.96
rs7229639	18q21.1	*SMAD7*	G	A	—	—	–	–	–	0.80	0.79	1.11	0.89–1.40	0.35	0.80	0.79	1.11	0.89–1.40	0.35
rs10411210	19q13.11	*RHPN2*	T	C	0.14	0.16	0.81	0.55–1.19	0.28	0.17	0.18	0.88	0.69–1.13	0.33	0.16	0.18	0.86	0.70–1.06	0.16
rs1800469	19q13.2	*B9D2, TGFB1*	G	A	0.54	0.51	1.15	0.88–1.50	0.32	0.51	0.54	0.87	0.73–1.05	0.15	0.52	0.53	0.95	0.82–1.11	0.52
rs2241714	19q13.2	*B9D2, TMEM91*	C	T	0.54	0.51	1.14	0.87–1.49	0.36	0.51	0.54	0.88	0.73–1.05	0.16	0.52	0.53	0.95	0.82–1.11	0.52
rs961253	20p12.3	*intergenic*	A	C	0.08	0.12	0.71	0.44–1.13	0.15	0.12	0.10	1.23	0.92–1.64	0.17	0.11	0.11	1.04	0.81–1.32	0.78
rs4813802	20p12.3	*intergenic*	G	T	0.23	0.21	1.14	0.82–1.58	0.43	—	—	—	—	—	0.23	0.21	1.14	0.82–1.58	0.43
rs2423279	20p12.3	*intergenic*	C	T	0.32	0.30	1.11	0.84–1.48	0.46	0.31	0.29	1.11	0.91–1.36	0.30	0.31	0.29	1.11	0.95–1.31	0.20
rs4925386	20q13.33	*LAMA5*	C	T	—	—	—	—	—	0.75	0.77	0.89	0.72–1.09	0.26	0.75	0.77	0.89	0.72–1.09	0.26

Abbreviations: SNP (single-nucleotide polymorphism), NCC (National Cancer Center), RAF (risk allele frequency), OR (odds ratio), CI (confidence interval), NCBI dbSNP (National Center for Biotechnology Information Database of Single Nucleotide Polymorphisms), CRC (colorectal cancer), and FDR (false discovery rate).

^a^A1 is risk and A2 is reference allele according to NCBI dbSNP.

^b^Additive effect by multivariate logistic regression model adjusted for sex. We compared CRC patients diagnosed at age <50 and ≥50 years (reference).

^c^Adjustments for multiple testing were not applied because associations were not statistically significant (FDR-adjusted *p*-value for rs704017 = 0.09).

^d^*P*-value for heterogeneity of rs704017 between NCC 2010–2013 and NCC 2000–2004 = 0.56.

**Table 3:** Associations between rs704017 and risk of colorectal cancer stratified by age groups (<50 and ≥50 years).

Genetic model^a^	NCC 2010–2013					NCC 2000–2004					Combined dataset				
	OR^b^	95% CI	*P*	*P*_heterogeneity_^c^	*P*_interaction_^d^	OR^b^	95% CI	*P*	*P*_heterogeneity_^c^	*P*_interaction_^d^	OR^b^	95% CI	*P*	*P*_heterogeneity_^c^	*P*_interaction_^d^
Additive model															
<50 years	0.87	0.63–1.19	0.37	0.04	0.04	0.84	0.65–1.07	0.15	0.21	0.19	0.83	0.69–1.01	0.06	0.02	0.02
≥50 years	1.24	1.06–1.45	6.5 × 10^−3^			1.01	0.88–1.10	0.90			1.08	0.97–1.19	0.15		
Dominant model															
<50 years	0.82	0.55–1.23	0.34	0.02	0.02	0.77	0.56–1.06	0.11	0.16	0.17	0.77	0.60–0.98	0.03	7.5 × 10^−3^	7.8 × 10^−3^
≥50 years	1.42	1.14–1.77	2.0 × 10^−3^			1.00	0.84–1.20	0.97			1.13	0.98–1.29	0.09		
Recessive model															
<50 years	0.88	0.45–1.73	0.71	0.45	0.47	0.88	0.51–1.52	0.66	0.62	0.53	0.88	0.58–1.34	0.55	0.47	0.48
≥50 years	1.17	0.85–1.60	0.33			1.03	0.77–1.38	0.82			1.05	0.85–1.29	0.68		

Abbreviations: NCC (National Cancer Center), OR (odds ratio), CI (confidence interval), and NCBI dbSNP (National Center for Biotechnology Information Database of Single Nucleotide Polymorphisms).

^a^Risk and reference allele were labeled according to NCBI dbSNP.

^b^Multivariate logistic regression model adjusted for age and sex stratified by age groups (<50 and ≥50 years).

^c^Cochran’s Q test.

^d^Wald test.

## References

[b1] Lichtenstein P. . Environmental and heritable factors in the causation of cancer—analyses of cohorts of twins from Sweden, Denmark, and Finland. N Engl J Med 343, 78–85, doi: 10.1056/NEJM200007133430201 (2000). 10891514

[b2] de la Chapelle A. Genetic predisposition to colorectal cancer. Nat Rev Cancer 4, 769–780, doi: 10.1038/nrc1453 (2004). 15510158

[b3] Welter D. . The NHGRI GWAS Catalog, a curated resource of SNP-trait associations. Nucleic Acids Res 42, D1001–1006, doi: 10.1093/nar/gkt1229 (2014). 24316577 PMC3965119

[b4] Stigliano V., Sanchez-Mete L., Martayan A. & Anti M. Early-onset colorectal cancer: a sporadic or inherited disease? World J Gastroenterol 20, 12420–12430, doi: 10.3748/wjg.v20.i35.12420 (2014). 25253942 PMC4168075

[b5] Tenesa A. & Dunlop M. G. New insights into the aetiology of colorectal cancer from genome-wide association studies. Nat Rev Genet 10, 353–358, doi: 10.1038/nrg2574 (2009). 19434079

[b6] Dozois E. J. . Young-onset colorectal cancer in patients with no known genetic predisposition - Can we increase early recognition and improve outcome? Medicine 87, 259–263, doi: 10.1097/MD.0b013e3181881354 (2008). 18794708 PMC4437192

[b7] Barault L. . Hypermethylator phenotype in sporadic colon cancer: study on a population-based series of 582 cases. Cancer Res 68, 8541–8546, doi: 10.1158/0008-5472.CAN-08-1171 (2008). 18922929

[b8] Jass J. R. Classification of colorectal cancer based on correlation of clinical, morphological and molecular features. Histopathology 50, 113–130, doi: 10.1111/j.1365-2559.2006.02549.x (2007). 17204026

[b9] Chang D. T. . Clinicopathologic and molecular features of sporadic early-onset colorectal adenocarcinoma: an adenocarcinoma with frequent signet ring cell differentiation, rectal and sigmoid involvement, and adverse morphologic features. Mod Pathol 25, 1128–1139, doi: 10.1038/modpathol.2012.61 (2012). 22481281

[b10] Tezcan G., Tunca B., Ak S., Cecener G. & Egeli U. Molecular approach to genetic and epigenetic pathogenesis of early-onset colorectal cancer. World J Gastrointest Oncol 8, 83–98, doi: 10.4251/wjgo.v8.i1.83 (2016). 26798439 PMC4714149

[b11] Ballester V., Rashtak S. & Boardman L. Clinical and molecular features of young-onset colorectal cancer. World J Gastroenterol 22, 1736–1744, doi: 10.3748/wjg.v22.i5.1736 (2016). 26855533 PMC4724605

[b12] Giraldez M. D. . Susceptibility genetic variants associated with early-onset colorectal cancer. Carcinogenesis 33, 613–619, doi: 10.1093/carcin/bgs009 (2012). 22235025

[b13] Zhang B. . Large-scale genetic study in East Asians identifies six new loci associated with colorectal cancer risk. Nat Genet 46, 533–542, doi: 10.1038/ng.2985 (2014). 24836286 PMC4068797

[b14] Cancer Genome Atlas N. Comprehensive molecular characterization of human colon and rectal cancer. Nature 487, 330–337, doi: 10.1038/nature11252 (2012). 22810696 PMC3401966

[b15] Forbes S. A. . COSMIC: mining complete cancer genomes in the Catalogue of Somatic Mutations in Cancer. Nucleic Acids Res 39, D945–950, doi: 10.1093/nar/gkq929 (2011). 20952405 PMC3013785

[b16] Constantinescu S. N., Girardot M. & Pecquet C. Mining for JAK-STAT mutations in cancer. Trends Biochem Sci 33, 122–131, doi: 10.1016/j.tibs.2007.12.002 (2008). 18291658

[b17] Xiong H. . Inhibition of JAK1, 2/STAT3 signaling induces apoptosis, cell cycle arrest, and reduces tumor cell invasion in colorectal cancer cells. Neoplasia 10, 287–297 (2008). 18320073 10.1593/neo.07971 PMC2259457

[b18] Peters U., Bien S. & Zubair N. Genetic architecture of colorectal cancer. Gut 64, 1623–1636, doi: 10.1136/gutjnl-2013-306705 (2015). 26187503 PMC4567512

[b19] Paparo L. . Differential expression of PTEN gene correlates with phenotypic heterogeneity in three cases of patients showing clinical manifestations of PTEN hamartoma tumour syndrome. Hered Cancer Clin Pract 11, 8, doi: 10.1186/1897-4287-11-8 (2013). 23886400 PMC3737036

[b20] Carder P. . Stabilised p53 facilitates aneuploid clonal divergence in colorectal cancer. Oncogene 8, 1397–1401 (1993). 8479757

[b21] Raman R. . Evidence for possible non-canonical pathway(s) driven early-onset colorectal cancer in India. Mol Carcinog 53 Suppl 1, E181–186, doi: 10.1002/mc.21976 (2014). 23168910 PMC3597761

[b22] Shuai K. & Liu B. Regulation of gene-activation pathways by PIAS proteins in the immune system. Nat Rev Immunol 5, 593–605, doi: 10.1038/nri1667 (2005). 16056253

[b23] Memmott R. M. . Phosphatidylinositol ether lipid analogues induce AMP-activated protein kinase-dependent death in LKB1-mutant non small cell lung cancer cells. Cancer Res 68, 580–588, doi: 10.1158/0008-5472.CAN-07-3091 (2008). 18199555 PMC3558831

[b24] Reich D. E. & Lander E. S. On the allelic spectrum of human disease. Trends Genet 17, 502–510 (2001). 11525833 10.1016/s0168-9525(01)02410-6

[b25] Balmain A., Gray J. & Ponder B. The genetics and genomics of cancer. Nat Genet 33 Suppl, 238–244, doi: 10.1038/ng1107 (2003). 12610533

[b26] Kim H. C., Kim C. N., Yu C. S., Roh S. A. & Kim J. C. Methylation of the hMLH1 and hMSH2 promoter in early-onset sporadic colorectal carcinomas with microsatellite instability. Int J Colorectal Dis 18, 196–202, doi: 10.1007/s00384-002-0445-0 (2003). 12673483

[b27] Liang J., Kalady M. F. & Church J. Young age of onset colorectal cancers. Int J Colorectal Dis 30, 1653–1657, doi: 10.1007/s00384-015-2341-4 (2015). 26358068

[b28] Kim Y., Jun J. K., Choi K. S., Lee H. Y. & Park E. C. Overview of the National Cancer screening programme and the cancer screening status in Korea. Asian Pac J Cancer Prev 12, 725–730 (2011). 21627372

[b29] Bacchus C. M. . Recommendations on screening for colorectal cancer in primary care. CMAJ, doi: 10.1503/cmaj.151125 (2016). PMC4786388 26903355

[b30] Qaseem A. . Screening for colorectal cancer: a guidance statement from the American College of Physicians. Ann Intern Med 156, 378–386, doi: 10.7326/0003-4819-156-5-201203060-00010 (2012). 22393133

[b31] Sung J. J. . Asia Pacific consensus recommendations for colorectal cancer screening. Gut 57, 1166–1176, doi: 10.1136/gut.2007.146316 (2008). 18628378

[b32] Choe J. W. . Screening colonoscopy in asymptomatic average-risk Koreans: analysis in relation to age and sex. J Gastroenterol Hepatol 22, 1003–1008, doi: 10.1111/j.1440-1746.2006.04774.x (2007). 17608845

[b33] Betes M. . Use of colonoscopy as a primary screening test for colorectal cancer in average risk people. Am J Gastroenterol 98, 2648–2654, doi: 10.1111/j.1572-0241.2003.08771.x (2003). 14687811

[b34] Chiu H. M. . A prospective study of the frequency and the topographical distribution of colon neoplasia in asymptomatic average-risk Chinese adults as determined by colonoscopic screening. Gastrointest Endosc 61, 547–553 (2005). 15812407 10.1016/s0016-5107(05)00121-5

[b35] Hassanzadeh J., Moradzadeh R., Rajaee Fard A., Tahmasebi S. & Golmohammadi P. A comparison of case-control and case-only designs to investigate gene-environment interactions using breast cancer data. Iran J Med Sci 37, 112–118 (2012). 23115440 PMC3470067

[b36] Shin A. . Isoflavone and Soyfood Intake and Colorectal Cancer Risk: A Case-Control Study in Korea. PLoS One 10, e0143228, doi: 10.1371/journal.pone.0143228 (2015). 26575841 PMC4648565

[b37] Zhang B. . Genome-wide association study identifies a new SMAD7 risk variant associated with colorectal cancer risk in East Asians. Int J Cancer 135, 948–955, doi: 10.1002/ijc.28733 (2014). 24448986 PMC4051826

[b38] Houlston R. S. . Meta-analysis of three genome-wide association studies identifies susceptibility loci for colorectal cancer at 1q41, 3q26.2, 12q13.13 and 20q13.33. Nat Genet 42, 973–977, doi: 10.1038/ng.670 (2010). 20972440 PMC5098601

[b39] Jia W. H. . Genome-wide association analyses in East Asians identify new susceptibility loci for colorectal cancer. Nat Genet 45, 191–196, doi: 10.1038/ng.2505 (2013). 23263487 PMC3679924

[b40] Cui R. . Common variant in 6q26-q27 is associated with distal colon cancer in an Asian population. Gut 60, 799–805, doi: 10.1136/gut.2010.215947 (2011). 21242260 PMC3095478

[b41] Tomlinson I. . A genome-wide association scan of tag SNPs identifies a susceptibility variant for colorectal cancer at 8q24.21. Nat Genet 39, 984–988, doi: 10.1038/ng2085 (2007). 17618284

[b42] Tenesa A. . Genome-wide association scan identifies a colorectal cancer susceptibility locus on 11q23 and replicates risk loci at 8q24 and 18q21. Nat Genet 40, 631–637, doi: 10.1038/ng.133 (2008). 18372901 PMC2778004

[b43] Zanke B. W. . Genome-wide association scan identifies a colorectal cancer susceptibility locus on chromosome 8q24. Nat Genet 39, 989–994, doi: 10.1038/ng2089 (2007). 17618283

[b44] Tomlinson I. P. . A genome-wide association study identifies colorectal cancer susceptibility loci on chromosomes 10p14 and 8q23.3. Nat Genet 40, 623–630, doi: 10.1038/ng.111 (2008). 18372905

[b45] Jiao S. . Genome-wide search for gene-gene interactions in colorectal cancer. PLoS One 7, e52535, doi: 10.1371/journal.pone.0052535 (2012). 23300701 PMC3530500

[b46] Study C. . Meta-analysis of genome-wide association data identifies four new susceptibility loci for colorectal cancer. Nat Genet 40, 1426–1435, doi: 10.1038/ng.262 (2008). 19011631 PMC2836775

[b47] Peters U. . Identification of Genetic Susceptibility Loci for Colorectal Tumors in a Genome-Wide Meta-analysis. Gastroenterology 144, 799–807 e724, doi: 10.1053/j.gastro.2012.12.020 (2013). 23266556 PMC3636812

