# Supplementary material for: Common risk variants for colorectal cancer: an evaluation of associations with age at cancer onset
Source: Sci Rep. 2017 Jan 13;7:40644. doi: 10.1038/srep40644 (PMC5233996; doi:10.1038/srep40644)
Supplement: Supplementary Tables [file srep40644-s1.doc]

**Common risk variants for colorectal cancer: an evaluation of associations with age at cancer onset**

**Nan Song^1^, Aesun Shin^1, 2, 3^, *^, †^, Ji Won Park^4, 5^, Jeongseon Kim^3^, Jae Hwan Oh^5, †^**

^1^Cancer Research Institute, Seoul National University College of Medicine, Seoul, 03080, Korea

^2^Department of Preventive Medicine, Seoul National University College of Medicine, Seoul, 03080, Korea

^3^Molecular Epidemiology Branch, National Cancer Center, Goyang, 10408, Korea

^4^Department of Surgery, Seoul National University College of Medicine and Hospital, Seoul, 03080, Korea

^5^Center for Colorectal Cancer, National Cancer Center, Goyang, 10408, Korea

*Corresponding authors

Tel: +82-2-740-8331; Fax: +82-2-747-4830; Email: [shinaesun@snu.ac.kr](mailto:shinaesun@snu.ac.kr)

Tel: +82-31-920-1535; Fax: +82-31-920-1148; Email: [jayoh@ncc.re.kr](mailto:jayoh@ncc.re.kr)

^†^These authors contributed equally to this work and should be considered as co-corresponding authors.

| Supplementary Table 1. Colorectal cancer susceptibility single-nucleotide polymorphisms previously identified by genome-wide association studies | | | | | | | |
| --- | --- | --- | --- | --- | --- | --- | --- |
| SNP | Cytogenetic  region | Chromosomal  Location | Mapped gene | Reported gene | Allele^a^ | | Reference |
|  |  |  |  |  | A1 | A2 |  |
| rs6687758 | 1q41 | 221991606 | *intergenic* | *DUSP10, intergenic* | G | A | Houlston et al. Nat Genet. 2010 |
| rs10936599 | 3q26.2 | 169774313 | *MYNN* | *MYNN, TERC, ACTRT3, LRRC34, intergenic* | T | C | Houlston et al. Nat Genet. 2010 |
| rs647161 | 5q31.1 | 135163402 | *C5orf66* | *PITX1* | A | C | Jia et al. Nat Genet. 2012 |
| rs7758229 | 6q25.3 | 160419220 | *SLC22A3* | *SLC22A3* | T | G | Cui et al. Gut. 2011 |
| rs6983267 | 8q24.21 | 127401060 | *CASC8, CCAT2* | *intergenic* | T | G | Tomlinson et al. Nat Genet. 2007 |
| rs7014346 | 8q24.21 | 127412547 | *CASC8* | *POU5FIP1, HsG57825, DQ515897* | G | A | Tenesa et al. Nat Genet. 2008 |
| rs10505477 | 8q24.21 | 127395198 | *CASC8* | *ORF, DQ515897, MYC* | G | A | Zanke et al. Nat Genet. 2007 |
| rs10795668 | 10p14 | 8659256 | *LOC105376400* | *intergenic* | A | G | Tomlinson et al. Nat Genet. 2008 |
| rs704017 | 10q22.3 | 79059375 | *ZMIZ1-AS1* | *AS1, ZMIZ1* | G | A | Zhang et al. Nat Genet. 2014 |
| rs11196172 | 10q25.2 | 112967084 | *TCF7L2* | *TCF7L2* | A | G | Zhang et al. Nat Genet. 2014 |
| rs1665650 | 10q25.3 | 116727589 | *HSPA12A* | *HSPA12A* | C | T | Jia et al. Nat Genet. 2012 |
| rs174537 | 11q12.2 | 61785208 | *MYRF* | *MYRF, FADS1, FADS2, FEN1* | T | G | Zhang et al. Nat Genet. 2014 |
| rs4246215 | 11q12.2 | 61796827 | *FEN1* | *FEN1, C11orf9, FADS1, FADS2* | T | G | Zhang et al. Nat Genet. 2014 |
| rs174550 | 11q12.2 | 61804006 | *FADS1* | *FADS1* | T | C | Zhang et al. Nat Genet. 2014 |
| rs1535 | 11q12.2 | 61830500 | *FADS2* | *FADS2, FEN FADS1* | A | G | Zhang et al. Nat Genet. 2014 |
| rs3802842 | 11q23.1 | 111300984 | *COLCA1, COLCA2* | *LOC120376, intergenic* | A | C | Tenesa et al. Nat Genet. 2008 |
| rs10849432 | 12p13.31 | 6276561 | *intergenic* | *PLEKHG6, TNFRSF1A, CD9* | T | C | Zhang et al. Nat Genet. 2014 |
| rs10774214 | 12p13.32 | 4259186 | *CCND2-AS1* | *CCND2, C12orf5, FGF6, RAD51AP1, FGF23, PARP11* | C | T | Jia et al. Nat Genet. 2012 |
| rs11169552 | 12q13.12 | 50761880 | *ATF1, LOC105369765* | *DIP2B, ATF1* | T | C | Houlston et al. Nat Genet. 2010 |
| rs7136702 | 12q13.13 | 50486433 | *intergenic* | *intergenic* | C | T | Jiao et al. PLoS One. 2012 |
| rs4444235 | 14q22.2 | 53944201 | *intergenic* | *BMP4* | C | T | Houlston et al. Nat Genet. 2008 |
| rs1957636 | 14q22.3 | 54093300 | *LOC105370507* | *-* | C | T | Zhang et al. Nat Genet. 2014 |
| rs4779584 | 15q13.3 | 32702555 | *intergenic* | *intergenic, GREM1, SCG5, CRAC1, HMPS* | C | T | Tomlinson et al. Nat Genet. 2008 |
| rs9929218 | 16q22.1 | 68787043 | *CDH1* | *CDH1* | A | G | Houlston et al. Nat Genet. 2008 |
| rs12603526 | 17p13.3 | 897353 | *intergenic* | *NXN* | C | T | Zhang et al. Nat Genet. 2014 |
| rs7229639 | 18q21.1 | 48924606 | *SMAD7* | *SMAD7* | G | A | Zhang et al. Nat Genet. 2014 |
| rs10411210 | 19q13.11 | 33041394 | *RHPN2* | *RHPN2* | T | C | Houlston et al. Nat Genet. 2008 |
| rs1800469 | 19q13.2 | 41354391 | *B9D2, TGFB1* | *TGFB1, B9D2* | G | A | Zhang et al. Nat Genet. 2014 |
| rs2241714 | 19q13.2 | 41363487 | *B9D2, TMEM91* | *TGFB1, B9D2* | C | T | Zhang et al. Nat Genet. 2014 |
| rs961253 | 20p12.3 | 6423634 | *intergenic* | *intergenic* | A | C | Houlston et al. Nat Genet. 2008 |
| rs4813802 | 20p12.3 | 6718948 | *intergenic* | *BMP2* | G | T | Peters et al. Gastroenterology. 2012 |
| rs2423279 | 20p12.3 | 7831703 | *intergenic* | *HAO1, PLCB1* | C | T | Jia et al. Nat Genet. 2012 |
| rs4925386 | 20q13.33 | 62345988 | *LAMA5* | *LAMA5, intergenic* | C | T | Houlston et al. Nat Genet. 2010 |
| Abbrevations: SNP (single-nucleotide polymorphism), MA (minor allele), NCBI (National Center for Biotechnology Information), and dbSNP (Database of Single Nucleotide Polymorphisms). | | | | | | | |
| ^a^A1 is risk and A2 is reference allele according to NCBI dbSNP. | | | | | | | |

| Supplementary Table 2. Allelic frequency comparison of identified susceptibility single-nucleotide polymorphisms between age of onset groups (<50 vs. ≥65 years) in colorectal cancer patients | | | | | | | | | | | | | | | |
| --- | --- | --- | --- | --- | --- | --- | --- | --- | --- | --- | --- | --- | --- | --- | --- |
| SNP | Cytogenetic  region | Mapped gene | Allele^a^ | | NCC 2010-2013 | | |  | NCC 2000-2004 | | |  | Combined dataset | | |
|  |  |  |  |  | OR^b^ | 95% CI | *P* |  | OR^b^ | 95% CI | *P* |  | OR^b^ | 95% CI | *P* |
|  |  |  | A1 | A2 |  |  |  |  |  |  |  |  |  |  |  |
| rs6687758 | 1q41 | *intergenic* | G | A | 0.91 | 0.61-1.35 | 0.63 |  | 1.03 | 0.82-1.30 | 0.81 |  | 0.99 | 0.81-1.21 | 0.90 |
| rs10936599 | 3q26.2 | *MYNN* | T | C | 0.97 | 0.68-1.39 | 0.87 |  | 1.03 | 0.83-1.28 | 0.81 |  | 1.02 | 0.85-1.23 | 0.84 |
| rs647161 | 5q31.1 | *C5orf66* | A | C | 1.06 | 0.74-1.52 | 0.76 |  | 1.17 | 0.94-1.44 | 0.16 |  | 1.15 | 0.96-1.38 | 0.13 |
| rs7758229 | 6q25.3 | *SLC22A3* | T | G | 0.99 | 0.67-1.48 | 0.98 |  | 0.89 | 0.69-1.14 | 0.35 |  | 0.93 | 0.76-1.15 | 0.51 |
| rs6983267 | 8q24.21 | *CASC8, CCAT2* | T | G | 0.94 | 0.68-1.31 | 0.73 |  | 0.93 | 0.75-1.14 | 0.46 |  | 0.92 | 0.77-1.10 | 0.37 |
| rs7014346 | 8q24.21 | *CASC8* | G | A | 0.99 | 0.70-1.40 | 0.96 |  | - | - | - |  | 0.99 | 0.70-1.40 | 0.96 |
| rs10505477 | 8q24.21 | *CASC8* | G | A | 0.93 | 0.68-1.29 | 0.68 |  | - | - | - |  | 0.93 | 0.68-1.29 | 0.68 |
| rs10795668 | 10p14 | *LOC105376400* | A | G | 1.15 | 0.82-1.62 | 0.42 |  | 1.10 | 0.88-1.37 | 0.42 |  | 1.12 | 0.93-1.35 | 0.23 |
| rs704017 | 10q22.3 | *ZMIZ1-AS1* | G | A | 0.63 | 0.44-0.90 | 0.01 |  | 0.80 | 0.64-1.01 | 0.06 |  | 0.77 | 0.63-0.93 | 6.6×10^-3^ |
| rs11196172 | 10q25.2 | *TCF7L2* | A | G | 1.35 | 0.89-2.04 | 0.16 |  | 0.96 | 0.76-1.21 | 0.73 |  | 1.05 | 0.86-1.29 | 0.64 |
| rs1665650 | 10q25.3 | *HSPA12A* | C | T | 0.86 | 0.60-1.25 | 0.44 |  | 1.01 | 0.81-1.27 | 0.90 |  | 0.98 | 0.81-1.18 | 0.82 |
| rs174537 | 11q12.2 | *MYRF* | T | G | 0.81 | 0.55-1.20 | 0.29 |  | 0.97 | 0.77-1.23 | 0.82 |  | 0.94 | 0.77-1.14 | 0.52 |
| rs4246215 | 11q12.2 | *FEN1* | T | G | - | - | - |  | 0.95 | 0.75-1.21 | 0.69 |  | 0.95 | 0.75-1.21 | 0.69 |
| rs174550 | 11q12.2 | *FADS1* | T | C | 1.21 | 0.82-1.80 | 0.33 |  | 1.04 | 0.82-1.32 | 0.75 |  | 1.07 | 0.88-1.31 | 0.51 |
| rs1535 | 11q12.2 | *FADS2* | A | G | 1.23 | 0.83-1.83 | 0.29 |  | 1.05 | 0.83-1.33 | 0.69 |  | 1.08 | 0.89-1.32 | 0.44 |
| rs3802842 | 11q23.1 | *COLCA1, COLCA2* | A | C | 1.07 | 0.76-1.50 | 0.70 |  | 0.99 | 0.80-1.22 | 0.90 |  | 1.00 | 0.84-1.20 | 0.96 |
| rs10849432 | 12p13.31 | *intergenic* | T | C | 0.75 | 0.49-1.33 | 0.17 |  | 1.11 | 0.84-1.47 | 0.46 |  | 0.97 | 0.77-1.22 | 0.82 |
| rs10774214 | 12p13.32 | *CCND2-AS1* | C | T | 0.83 | 0.59-1.17 | 0.29 |  | 0.92 | 0.74-1.15 | 0.47 |  | 0.91 | 0.76-1.09 | 0.30 |
| rs11169552 | 12q13.12 | *ATF1, LOC105369765* | T | C | 0.84 | 0.58-1.21 | 0.34 |  | 1.12 | 0.90-1.40 | 0.31 |  | 1.03 | 0.85-1.24 | 0.80 |
| rs7136702 | 12q13.13 | *intergenic* | C | T | 0.83 | 0.59-1.17 | 0.28 |  | 1.06 | 0.86-1.31 | 0.56 |  | 0.98 | 0.82-1.17 | 0.85 |
| rs4444235 | 14q22.2 | *intergenic* | C | T | 1.17 | 0.85-1.62 | 0.32 |  | 0.96 | 0.78-1.19 | 0.71 |  | 1.01 | 0.85-1.21 | 0.90 |
| rs1957636 | 14q22.3 | *LOC105370507* | C | T | 0.97 | 0.69-1.36 | 0.86 |  | - | - | - |  | 0.97 | 0.69-1.36 | 0.86 |
| rs4779584 | 15q13.3 | *intergenic* | C | T | 0.88 | 0.54-1.43 | 0.61 |  | 1.02 | 0.76-1.36 | 0.91 |  | 0.96 | 0.75-1.23 | 0.75 |
| rs9929218 | 16q22.1 | *CDH1* | A | G | 0.73 | 0.46-1.14 | 0.17 |  | 1.05 | 0.76-1.45 | 0.76 |  | 0.95 | 0.73-1.23 | 0.69 |
| rs12603526 | 17p13.3 | *intergenic* | C | T | 1.09 | 0.76-1.58 | 0.63 |  | - | - | - |  | 1.09 | 0.76-1.58 | 0.63 |
| rs7229639 | 18q21.1 | *SMAD7* | G | A | - | - | - |  | 1.05 | 0.81-1.36 | 0.70 |  | 1.05 | 0.81-1.36 | 0.70 |
| rs10411210 | 19q13.11 | *RHPN2* | T | C | 0.79 | 0.49-1.27 | 0.34 |  | 1.00 | 0.76-1.33 | 0.99 |  | 0.93 | 0.73-1.19 | 0.58 |
| rs1800469 | 19q13.2 | *B9D2, TGFB1* | G | A | 1.16 | 0.82-1.63 | 0.40 |  | 0.86 | 0.70-1.05 | 0.14 |  | 0.93 | 0.78-1.10 | 0.38 |
| rs2241714 | 19q13.2 | *B9D2, TMEM91* | C | T | 1.18 | 0.84-1.67 | 0.34 |  | 0.86 | 0.70-1.06 | 0.15 |  | 0.93 | 0.78-1.11 | 0.43 |
| rs961253 | 20p12.3 | *intergenic* | A | C | 0.82 | 0.46-1.46 | 0.51 |  | 1.41 | 1.00-1.99 | 0.05 |  | 1.21 | 0.90-1.62 | 0.21 |
| rs4813802 | 20p12.3 | *intergenic* | G | T | 0.90 | 0.60-1.35 | 0.61 |  | - | - | - |  | 0.90 | 0.60-1.35 | 0.61 |
| rs2423279 | 20p12.3 | *intergenic* | C | T | 1.12 | 0.77-1.64 | 0.55 |  | 1.07 | 0.85-1.35 | 0.54 |  | 1.09 | 0.90-1.33 | 0.38 |
| rs4925386 | 20q13.33 | *LAMA5* | C | T | 1.12 | 0.77-1.64 | 0.55 |  | 0.90 | 0.71-1.15 | 0.40 |  | 0.90 | 0.71-1.15 | 0.40 |
| Abbreviations: SNP (single-nucleotide polymorphism), RAF (risk allele frequency), OR (odds ratio), CI (confidence interval), and NCBI dbSNP (National Center for Biotechnology Information Database of Single Nucleotide Polymorphisms). | | | | | | | | | | | | | | | |
| ^a^A1 is risk and A2 is reference allele according to NCBI dbSNP. | | | | | | | | | | | | | | | |
| ^b^Additive effect by multivariate logistic regression model adjusted for sex. We compared colorectal cancer patients diagnosed at age <50 and ≥50 years (reference). | | | | | | | | | | | | | | | |

| Supplementary Table 3. Genotype frequency of rs704017 between colorectal cancer cases and controls stratified by age groups (<50 and ≥50 years) | | | | | | | | | | | | | | |
| --- | --- | --- | --- | --- | --- | --- | --- | --- | --- | --- | --- | --- | --- | --- |
| Genotype | NCC 2010-2013 | | | |  | NCC 2000-2004 | | | |  | Combined set | | | |
|  | Case | | Control | |  | Case | | Control | |  | Case | | Control | |
|  | N | (%) | N | (%) |  | N | (%) | N | (%) |  | N | (%) | N | (%) |
| Rs704017 (<50 years) |  |  |  |  |  |  |  |  |  |  |  |  |  |  |
| AA | 62 | (45.9) | 125 | (41.1) |  | 150 | (51.0) | 136 | (44.3) |  | 212 | (49.4) | 261 | (53.0) |
| AG | 60 | (44.4) | 146 | (48.0) |  | 117 | (39.8) | 140 | (45.6) |  | 177 | (41.3) | 286 | (46.8) |
| GG | 13 | (9.6) | 33 | (10.9) |  | 27 | (9.2) | 31 | (10.1) |  | 40 | (9.3) | 64 | (10.5) |
| Rs704017 (≥50 years) |  |  |  |  |  |  |  |  |  |  |  |  |  |  |
| AA | 182 | (36.5) | 465 | (45.2) |  | 437 | (43.4) | 454 | (44.7) |  | 619 | (41.1) | 919 | (45.0) |
| AG | 246 | (49.3) | 438 | (42.6) |  | 457 | (45.4) | 454 | (44.7) |  | 703 | (46.7) | 892 | (43.6) |
| GG | 71 | (14.2) | 126 | (12.2) |  | 113 | (11.2) | 107 | (10.5) |  | 184 | (12.2) | 233 | (11.4) |
| Abbreviations: NCC (National Cancer Center). | | | | | | | | | | | | | | |
